# Exploring the Role of App Features in Providing Continuity of Care to Users on a Digital Mental Health Platform (Wysa): Retrospective Mixed Methods Observational Study

**DOI:** 10.2196/73033

**Published:** 2026-01-26

**Authors:** Chaitali Sinha, Riddhi Thakkar, Saha Meheli, Dyuthi Dinesh

**Affiliations:** 1 Wysa Inc, 131 Dartmouth Street #3, Boston, MA, 02116, United States

**Keywords:** Wysa, continuity of care, relational continuity, digital mental health interventions, DMHI, conversational agent, mental health coaches, artificial intelligence, AI

## Abstract

**Background:**

Despite digital mental health services growing at a rapid pace to address global mental health needs, there exist challenges of low engagement and attrition. Ensuring continuity of care in the digital context can positively impact mental health care delivery and adherence to treatment, helping to establish digital mental health interventions (DMHIs) as a viable option for mental health support.

**Objective:**

This study aimed to examine the impact of adjunct app features of the mental health app Wysa and their ability to promote engagement and adherence to the text-based coaching sessions.

**Methods:**

This retrospective mixed methods observational study was based on real-world app data from users (n=1213) who subscribed to text-based sessions with mental health coaches (MHCs) between February 1 and July 31, 2022. Their engagement with the adjunct app features, such as brief interventions with the conversational agent, self-management tools, and journaling, was analyzed quantitatively using descriptive statistics. Acceptability of the app features was also assessed using qualitative feedback data. Adherence to sessions with MHCs was compared between app feature users (n=1042, 85.9%) and nonfeature users (n=171, 14.1%) using inferential statistics. Subgroup analysis was not feasible in the absence of demographic and clinical user data, potentially limiting the generalizability of the findings.

**Results:**

Findings demonstrated high use of the adjunct app features, which allowed communication with the MHCs in between sessions. The thematic analysis captures user experiences of helpfulness within the app and with the MHCs. The Mann-Whitney U test indicated that users who accessed one or more features completed significantly more sessions compared with users who did not use any feature (Mann-Whitney U=154,085.0; *P*<.001; r_B_=0.73) with a large effect size. The odds ratio analysis indicated that users were almost thrice as likely to complete sessions after using the adjunct app features (odds ratio 2.91, 95% CI 2.24-3.38; *P*<.001).

**Conclusions:**

Inclusion of adjunct app features enhances continuity in care delivery between sessions with MHCs and is associated with improved engagement with DMHIs. Further efforts are needed to assess the impact of this approach in DMHIs on clinical mental health outcomes.

## Introduction

The global burden of mental health disorders has increased significantly over the past decade. The World Health Organization (WHO) estimates that about 970 million people were living with a mental disorder in 2019 [1]. Availability of mental health services remains widely inaccessible, unaffordable, and inadequate. Social stigma around mental health disorders further widens the gap between access and care, leaving a significant number of people without timely and adequate mental health support [2]. Studies estimate that only a third of people with depressive disorders receive formal mental health care globally, signifying the urgent need for innovation to offer timely support and equitably deliver appropriate care [3].

Health care access and delivery systems create a complex environment for patients to navigate, and this complexity adversely affects continual access to necessary care and overall health outcomes [4]. A cohesive, interconnected, and efficient system is especially necessary to address the care needs of patients with complex physical and mental health challenges [5,6]. Continuity of care, as a concept, focuses on a patient-centered approach to enhance the quality of care delivered by coordinated services [7-9]. It is the support that an individual receives throughout the process of care delivery, within and outside a consultation session with a health professional [7]. This can be bridged by allied health professionals, social support systems, and digital technologies. Health care providers and patients emphasize the role of continuity in decreased symptom severity and enhanced health outcomes [5,10].

In mental health, continuity of care is often linked to the therapeutic alliance between the health care provider and the patient. A meta-analytic review in mental health found a moderate effect size in the relationship between clinical outcomes and the therapeutic alliance [11], which relies on the ability to access necessary information, maintain an ongoing patient-therapist collaboration, and have access to self-management options [12,13].

Digital mental health interventions (DMHIs) have emerged as a compelling solution to bridge the continuity gap due to their ability to increase access across geography and time of day [14]. They offer users low-cost support at their convenience, without requiring appointments or transportation [15-17]. DMHIs have been studied separately to examine their ability to improve mental health outcomes, but their impact on parallel engagement with mental health coaching has not been examined.

This mixed methods retrospective observational study examines this ecosystem of care using real-world data from the mental health app Wysa (Wysa Inc) to explore the following objectives: (1) to assess the use and acceptability of the adjunct app features between sessions among users who had subscribed to mental health coaching on a text-based platform and (2) to determine whether higher use of adjunct app features leads to improved engagement and adherence to sessions with mental health coaches (MHCs).

## Methods

### App Background

Wysa is a digital mental health app that has been publicly available since 2016. The app’s direct-to-consumer version is anonymous for users and consists of an artificial intelligence (AI)–enabled conversational agent (CA), weekly sessions with MHCs, asynchronous messaging with MHCs, and cognitive behavioral therapy (CBT)–based brief interventions. All Wysa CA resources have been clinically validated by licensed mental health professionals.

The features within this app are not a replacement for face-to-face psychotherapy and are not used for diagnosis, prognosis, treatment, or to provide any type of state-regulated mental health services in the client’s country of residence. Users who expressed suicidality or self-harm were signposted to appropriate helpline services. The Wysa CA is free for all users, while access to certain tools and sessions with the MHCs is offered as paid features.

### Wysa App Features

#### Mental Health Coaching

This service connects users to qualified mental health and well-being professionals, who have undergone standardized in-house training to provide legal, ethical, and clinically safe psychological support and mental health care. The MHCs are psychologists with master’s- or doctoral-level qualifications. Each session with an assigned MHC is scheduled for 30 to 45 minutes and is conducted through a live text-based messaging system on the platform.

#### Adjunct App Features

##### Asynchronous Communication With MHC (Journaling)

Users who subscribe to sessions with MHCs also have the option to send messages to their assigned MHC in between scheduled sessions to receive continued support. The MHC typically responds within 24 hours.

##### Conversational Agent

Wysa, an AI-enabled CA, provides a digital therapeutic space for users to receive brief evidence-based CBT interventions and access guided listening. User conversations occur with a free-text CA supported by models developed using natural language processing and natural language understanding. The CA also provides users with self-management tools that leverage evidence-based therapy techniques including CBT, acceptance and commitment therapy, and mindfulness-based approaches. The tools cover a range of interventions that support users through difficulties such as anxiety, depression, anger management, and sleep-related concerns, among others.

### Ethical Considerations

Wysa’s privacy policy and terms of service strictly ensure the anonymity and privacy of its users. Users could directly access the app without registration, with no personally identifying information collected during app use. Furthermore, any inadvertently shared personally identifying information (such as name, nationality, location, gender, etc) was redacted from the backend. Thus, only anonymized data were used in the research. An independent researcher reproduced all primary reported results and validated the reported findings to minimize any impact of the conflict of interest.

The study protocol was reviewed by Pearl Institutional Review Board (Indianapolis, Indiana, United States) and determined to be exempt from institutional review board oversight as it involved retrospective analysis of deidentified user data (exemption determination: 2025‐0025, 01/17/2025).

### Study Design and Sampling

This study followed a mixed methods retrospective observational design to examine the real-world usage, engagement, and feedback data of anonymized global users of Wysa from February 1 to July 31, 2022. The inclusion criteria for this study were users who subscribed to text-based sessions with MHCs during the study period. As the dataset consisted of anonymized user records, no additional inclusion or exclusion criteria were applied, and the sample was considered representative of Wysa’s global user base. This resulted in a sample of 1213 users. A total of 1042 (85.9%) users used at least 1 adjunct app feature, whereas 171 (14.1%) users did not use any app features between sessions with their MHCs during the study period.

### Data Analysis

#### Objective 1: Use and Acceptability of Sessions With MHCs and Adjunct App Features

The use metrics of the features (sessions and tools used with the CA, journaling frequency, and sessions with MHCs) were analyzed using descriptive statistics. The acceptability of these features and sessions with the MHCs was quantified using a helpfulness rating and also assessed qualitatively by conducting a thematic analysis [18] of feedback provided by the users. Thematic coding was performed independently by 2 researchers, with discrepancies resolved through discussion to enhance reliability. This was initiated by the generation of preliminary codes, which were then grouped into potential subthemes and themes. The data were then verified for relevance to the themes at each level, and the initial set of themes was verified in relation to the coded extracts. The themes were finalized on the basis of their relevance to the objective.

#### Objective 2: Impact of the Adjunct App Features on Engagement and Adherence With MHC Sessions

Inferential statistics were used to examine whether the adjunct app features improved engagement and adherence to MHC sessions. The analysis compared users who used at least 1 adjunct app feature with users who accessed sessions with MHCs without using any other app feature. Normal distribution of the data was assessed using the Shapiro-Wilk test for normality, and as the data were not normally distributed, the Mann-Whitney U test was used to measure groupwise differences in completion of sessions with MHCs between feature users (1042/1213, 85.9%) and the comparative group of users who did not use features (171/1213, 14.1%). Furthermore, an odds ratio (OR) analysis was conducted to explore whether using the adjunct app features (exposure) resulted in completion or noncompletion of the subsequent session with the MHC (outcome).

## Results

### Objective 1: Use at and Acceptability of Adjunct App Features and Sessions With the MHCs

#### Sessions With MHCs

For app feature users (n=1042), a total of 10,116 sessions were scheduled with MHCs on the app between February 1 and July 31, 2022. Of these, 6854 (68.4%) sessions were completed, and 3162 (31.6%) were either missed or canceled. Users who did not use any other app features (n=171) scheduled 243 sessions, completed 94 (38.4%) sessions, and missed or canceled 149 (61.3%) sessions during the study period. App feature users, on average, scheduled 9.61 (SD 10.99) and completed 6.57 (SD 8.20) sessions compared with non–app feature users, who scheduled and completed an average of 1.42 (SD 1.41) and 0.55 (SD 0.96) sessions, respectively. The overall data of scheduled sessions across both groups indicated a right-skewed distribution with a positive skewness of 2.13.

The user-rated helpfulness of MHC sessions across both groups was assessed using quantitative user feedback from 7823 sessions obtained on a scale of 1 to 3, out of which 6869 (87.8%) sessions were found to be very helpful. Among adjunct app feature users, 0.9% (69/7620) found the MHC sessions to be not helpful compared with 3.95% (8/203) of nonfeature users.

#### Use of Adjunct App Features

##### Asynchronous Communication With MHC (Journaling)

Out of 1042 app feature users, the journaling feature that enabled asynchronous communication with MHCs was used at least once by 813 (78%) users, who sent an average of 136.38 (SD 296.06) messages per user over the study period and 14.86 (SD 20.81) messages per user per journaling session. Some users provided feedback that this feature gave them opportunities to express their thoughts and emotions beyond the sessions. One stated how their “sessions are very helpful whenever (they) use the journal.”

##### AI-Led Tools Through the CA

Out of 1042 app feature users, 946 (90.8%) used the CA feature in this period. The average rating given to the CA was 3.86 out of 5, largely indicating positive feedback. Additionally, 29 users (2.4%) provided qualitative feedback, commenting on the helpfulness of the CA. For instance, one user said, “Wysa helps me see the brighter side of things.” Many users also highlighted the therapeutic relationship, commenting that the CA was “comforting” and “helpful” in rethinking and “breaking negative thought cycles.” However, few users also felt that the CA was “too robotic,” “limited,” and “not helpful.” The most used tools delivered by the CA were the mindfulness-based interventions, sleep, and stress management tools. One of the users shared, “I liked the idea of having tools and steps to help me move forward with my goals as well as helping me cope with unwanted isolation.”

### Qualitative Feedback on MHC Sessions

User feedback on the helpfulness of MHC sessions was overwhelmingly positive, with 87.8% (6869/7823) of sessions rated as very helpful across both groups. The thematic analysis of qualitative feedback (n=959) on Wysa’s MHC sessions provided by 322 (30.9%) out of 1042 users yielded 2 major themes: quality of services and personal growth and development.

#### Access to Support and Safe Spaces

Users positively described the quality of services and continued *support received* from Wysa’s MHCs and the *establishment of an emotionally safe space through the app*.

Users often felt truly understood and attributed their progress to their MHCs, whom they described as caring, trustworthy, and compassionate. A user emphasized, “My therapist has helped me make huge progress in my capacity to handle life and difficult situations, and I am constantly learning from her. Her support has made a big difference in my life.”

A user also stated that it is “great to have a safe space where I can share my thoughts and feelings,” while another user expressed their gratitude for a trusting space for expression and emotional regulation: “Thank you. It’s actually difficult to open up about these things and thank you for this very safe space to do so.”

However, some users also expressed discomfort with the text-based form of MHC sessions and noted difficulty in communicating at times with their assigned MHCs. A user wrote, “Lag with texting and the responses were slow*.*”

#### Personal Growth and Development

The users also attributed personal growth and development to the MHC sessions, stating that these sessions helped improve self-esteem, confidence, and skill building. One user reported, “This session really helped me refocus on strengths and rebuild my self-esteem.” Another user emphasized how MHC sessions were “helping me recognize my wins and that I am able to calm myself down and make rational decisions really helped boost my confidence in myself*.*”

A positive response to MHC support was also noted, as users reported enhanced self-awareness and learning skills, such as goal setting, reframing dysfunctional thoughts, and adopting diverse perspectives for actions and behaviors. One user described learning to “see the kind of harshness I project towards myself.” Users also emphasized the benefits of using tools and techniques recommended by their MHCs. One user mentioned, “I liked the idea of having tools and steps to help me move forward with my goals as well as helping me cope with unwanted isolation.” In some cases, users also specifically asked for *goal-oriented approaches*. One user stated, “I feel like we should focus on coping skills.”

Furthermore, users also highlighted specific requests and advice they sought from their MHCs, such as “some tips for building boundaries before my next shift,” “discuss ways to improve my time management,” or “more concrete plans that I can act on.” This indicated the specific types of personalized and continual care and support the users sought from their trusted MHCs.

### Objective 2: Impact of the Adjunct Features on Engagement and Adherence

The number of sessions completed with MHCs among app feature user and nonfeature user groups was right skewed, with a positive skewness of 1.95 and 3.91, respectively. The Mann-Whitney U test indicated that the adjunct app feature users (1042/1213, 85.9%; mean 6.57, SD 8.2) completed significantly more sessions (mean difference 6.02; Mann-Whitney U=154,085.0; *P*<.001; r_B_=0.73) with a large effect size, compared with users who did not use any features (171/1213, 14.1%; mean 0.55, SD 0.95).

Results from the OR analysis indicate that the odds of completing the subsequent session increased significantly when users used any of the adjunct features (OR 2.91, 95% CI 2.24-3.38; *P*<.001). Thus, users were almost 3 times as likely to complete their next session with their MHC after using at least one of the app features than when they did not use any feature in between sessions.

## Discussion

### Principal Findings

This retrospective observational study explores the use of a DMHI, Wysa, using real-world data to understand its association with improving user adherence and engagement with MHC sessions. The study results indicate that users who engaged with the chatbot and its integrated brief interventions are significantly more likely to engage with subsequent MHC sessions compared with those who do not use any adjunct app features. These findings support recent studies where patients have demonstrated a higher level of acceptability and readiness to access help after the use of a brief digital intervention [19,20].

DMHIs are a scalable solution that supports the effective expansion of access to mental health resources to wider populations [21] and can enhance relational continuity within care provision [22]. Previous studies have found that human support, text-based communication [23,24], integration of conversational agents [25], and self-management tools [26] can facilitate the development of a therapeutic alliance, improve continuity of care, and increase engagement with the DMHIs [22].

Asynchronous journaling with the MHCs, a text-based messaging feature, was used the most, providing insight into the need to be able to communicate outside of the session. It has been suggested that the features supporting text-based communications are an effective means of maintaining continued contact between patients and primary care providers, which enables timely exchange of vital information and needs and develops a strong working alliance [27,28].

The high use of self-management tools has been seen to provide a holistic care environment and promote a sense of agency and responsibility among patients [29]. Self-management tools in mental health care have been established as a cost-effective resource [30] that improves overall patient outcomes [31] when offered as an adjunct to standard-of-care therapies. Our study corroborates these findings, showing that users notably engage with self-management tools and that integrating them in DMHIs is a vital component in establishing a sustained therapeutic relationship.

More recently, AI-based CAs have emerged as a compelling solution that can provide a sense of constant presence and comfort, as they help in integrating disparate aspects of a digital platform [32,33]. A CA-monitored user interface can be a personalized tool that provides relevant information, self-management resources, reminders for adherence, and a platform to effectively monitor symptoms to support continuous care delivery to patients [34,35]. Similarly, high patient uptake and the use of Wysa’s AI-based CA were also noted in our study and were associated with improved overall engagement with the MHC sessions, suggesting its ability to support development of a therapeutic alliance. A meta-analysis noted that additional factors correlated with high engagement in DMHIs include gender, higher socioeconomic status, lower symptom severity, and education [36].

Improving user engagement and ensuring continuity of care is an essential factor to maximize the potential of DMHIs [37,38]. Research conducted in traditional mental health services also finds that providing resources that enhance continuity of care between treatment sessions improves engagement and adherence to overall treatment cycles and reduces the need for frequent hospital visits [39-43]. Improved relational continuity in traditional care delivery settings has also been associated with better overall mental health outcomes and a reduction in symptom severity in patients with severe mental health and psychiatric disorders, further highlighting the necessity of also integrating the construct in scalable digital mental health care delivery mechanisms [44,45].

Therefore, this study has important implications for the design and development of DMHIs as well as the broader mental health services. It highlights the need to consider a holistic approach and an overall quality of support while designing digital interventions to deliver mental health care. As demonstrated in this study, adjunct features such as evidence-based self-management tools, AI-based conversational agents, and journaling features can be some effective tools to provide continuous support that can be leveraged to improve patient adherence and engagement with the app-based DMHIs, along with other correlated factors. These features can be associated with sustained therapeutic relationships between patients and their MHCs in a digital environment and support client empowerment [43]. Thus, DMHIs could be designed to incorporate features based on similar principles to increase their effectiveness, improve user satisfaction, and maximize their application as an effective task sharing tool. Future research should further investigate the effectiveness of tools and factors in enhancing continued care delivery systems in the context of digital mental health and the impact of improving continuity of care on clinical mental health outcomes.

### Strengths and Limitations

To our knowledge, this study is unique in its approach of using real-world evidence to examine whether provision of certain adjunct app features in DMHIs is associated with building therapeutic relationships and improving overall patient engagement with mental health apps. Moreover, the approach draws its strength from the objectivity of its methodology. The retrospective observational approach enables examination of real-world user behaviors. The qualitative feedback reported further adds to the richness of information by providing nuanced user perspectives. In addition, potential user selection bias was minimized by applying a broad inclusion criteria and no exclusion criteria to the anonymized user data. However, the study also has several limitations. First, we were unable to measure the impact of improved continuity of care on differences in clinical efficacy and changes in symptom severity due to limitations of the dataset. Additionally, we could not measure any meaningful differences between demographic or clinical characteristics between app feature users and nonfeature users due to the retrospective design of the study, which limits the generalizability of the findings. Finally, another methodological constraint arising from the retrospective design was the uneven distribution and missingness of the data between app feature users and nonfeature users that limited the ability to triangulate the findings in this study.

However, this research lays the groundwork for more comprehensive exploration of continuity of care within the context of DMHIs. Therefore, future research could examine how providing adjunct features on a mental health app can impact overall engagement and further improve the clinical mental health outcomes for individuals, along with comparative investigations into the effectiveness of different adjunct features in increasing engagement and adherence. Subsequent studies can also delve into the experiences and perspectives of health care providers and users, along with prospective trials to explore feature use that enables continuity in the care delivered. Furthermore, DMHI services can also benefit from research using standardized methods for assessing continuity of care in the digital context, which in turn requires further investigation and guidelines.

To successfully scale mental health services to meet growing public mental health demands, it is necessary to build comprehensive digital mental health platforms that encompass various services such as coaching services, provide personalized knowledge and information, and support self-management [14,46]. The positive associations between developing relational continuity and improved clinical outcomes for individuals with mental health concerns underscore the importance of ongoing efforts to enhance care continuity in the digital context [6].

This study has used the STROBE (Strengthening the Reporting of Observational studies in Epidemiology) checklist for observational studies to enhance clarity and reproducibility of the findings (Checklist 1) [47].

### Conclusions

The use of DMHIs as a powerful tool to expand access to MHCs and services has increased substantially. Despite methodological constraints, this study provides foundational evidence that integrating additional features, such as AI-enabled CAs, self-management tools, and free communication platforms, among other factors, can support improvements in engagement and adherence to DMHIs and the delivery of continued support to patients outside of sessions with MHCs. Further exploration and evidence are needed to assess factors that improve the effectiveness of DMHIs through increased engagement, support a strong therapeutic relationship, enable a holistic digital care environment, and subsequently improve clinical mental health outcomes.

## Supplementary material

10.2196/73033 Checklist 1 STROBE checklist.

## Abbreviations

AI: artificial intelligence
CA: conversational agent
CBT: cognitive behavioral therapy
DMHI: digital mental health intervention
MHC: mental health coach
OR: odds ratio
STROBE: Strengthening the Reporting of Observational studies in Epidemiology
WHO: World Health Organization
